# Omics-CNN: A comprehensive pipeline for predictive analytics in quantitative omics using one-dimensional convolutional neural networks

**DOI:** 10.1016/j.heliyon.2023.e21165

**Published:** 2023-10-28

**Authors:** Anastasia Zompola, Aigli Korfiati, Konstantinos Theofilatos, Seferina Mavroudi

**Affiliations:** a Department of Electrical and Computer Engineering, University of Patras, Patras, Greece; b InSyBio PC, Patras Science Park, Patras, Greece; c King's British Heart Foundation Centre, Kings College London, United Kingdom; d Department of Nursing, School of Rehabilitation Sciences, University of Patras, Patras, Greece

**Keywords:** Convolutional neural networks, Transcriptomics, Personalized medicine, Covid-19, Ischemic stroke

## Abstract

**Background and objective:**

The development of machine learning-based models that can be used for the prediction of severe diseases has been one of the main concerns of the scientific community. The current study seeks to expand a highly sophisticated tool, the Convolutional Neural Networks, making it applicable in multidimensional omics data classification problems and testing the newly introduced method on publicly available transcriptomics and proteomics data.

**Methods:**

In this study, we introduce Omics-CNN, a Convolutional Neural Network-based pipeline, which couples Convolutional Neural Networks with dimensionality reduction, preprocessing, clustering, and explainability techniques to make them suitable to build highly accurate and interpretable classification models from high-throughput omics data. The developed tool has the potential to classify patients depending on the expression of genetic and clinical factors and identify features that can act as diagnostic biomarkers. Regarding dimensionality reduction, univariate and multivariate techniques were explored and compared. Gradient Weighted Class Activation Mapping analysis was performed to determine the most important features in the classification of the samples after training the model.

**Results:**

The newly introduced pipeline was applied to one transcriptomics and one proteomics dataset for the identification of diagnostic models and biosignatures for Ischemic Stroke (IS) and COVID-19 infection, reporting highly accurate biosignatures with accuracies of 96 % and 95.41 %, respectively. Meanwhile, classification models based solely on a small part of attributes provided lower predictive accuracy, but identified compact transcript biosignature (KRT15, VPRBP, TNFRSF4, GORASP2) for Ischemic Stroke and protein biosignature (ADGRB3, VNN2, AGER, CIAPIN1) for Covid-19 infection diagnosis, respectively.

**Conclusions:**

Omics-CNN, overcame the inherent problems of applying Convolutional Neural Networks for the training diagnostic models with quantitative omics data, outperforming previous models of machine learning developed using the same datasets for Ischemic Stroke and Covid-19 infection diagnosis, determining the most contributing biomarkers for both diseases.

## 1 Introduction

The big data revolution has started being noticeable in the field of Biology. High-throughput technologies (e.g., microarrays and next-generation sequencing) produce large amounts of biological data also known as omics. Omics include different types of data, such as genomics, epigenomics, transcriptomics, and proteomics, and can contribute to the comprehensive understanding of biological processes and pathways as well as promote the understanding of life-threatening diseases. The combination of such information lays the groundwork for precision medicine. Precision medicine uses data from different sources, thus offering a holistic description of a patient's overall health expediting disease diagnosis, and allowing for the selection of the most suitable therapeutic protocol for each patient [1]. The increased complexity of omics data in terms of dimensionality and noise impedes their analysis with simple statistical tests. As a result, omics data demand more sophisticated methods of computational intelligence, which have the capability of capturing complex nonlinear and hierarchical associations between the examined features.

In this context, Deep Neural Networks [2] are powerful computing tools that utilize the high correlation of the input data in sample classification. In recent years such computing tools have attracted interest in the analysis of complex data, including omics data. Indicatively some of the studies that have been conducted involve complicated architectures, which either combine different types of Deep Neural Networks, such as Recurrent Neural Networks with Convolutional Neural Networks [3] or analyze different data, such as clinical or multi-omics data [4]. There is a clear trend toward incorporating high-throughput omics or multi-omics analysis in biomedical research to explain the complex relationships between molecular layers. Such powerful tools can be widely adapted to various applications of high-dimensional omics data and have great potential to facilitate more accurate and personalized clinical decision-making, especially in life-threatening diseases, such as cancer, metastasis of cancer [5], stroke [6], or Covid-19 [7].

Convolutional Neural Networks (CNNs) have been widely used for classification and regression applications using imaging data but despite their feature of taking into consideration complex relationships between the utilized inputs which is very suitable for the analysis of omics data, only a few applications have been conducted on omics data due to technical limitations. In Ref. [8], the authors developed a model called, OmicsMapNet for the analysis of high-dimensional omics data as 2-dimensional images. The most contributory features in the trained CNN were confirmed in pathway analysis. Another previous study [9] proposed a CNN approach that combines spectral clustering information processing to classify lung cancer, with greater performance than other machine learning algorithms, such as Support Vector Machines (SVM) or Random Forests (RFs). Moreover, as the power of gene expression profile in cancer identification has been proven, in another conducted analysis [10], 2D images were generated, by integrated gene expression profiles and protein-protein interaction (PPI) network from human samples, to be analyzed by a CNN. Another approach applying a CNN to non-image data [11], called DeepFeature, was used to successfully transform omics data into a form that is optimal for fitting a CNN model and returned sets of the most important genes used internally for computing predictions.

Despite the promising results of these applications, CNN methods are not generally applicable to omics data because of the significantly higher number of features compared to the available samples in most of these datasets and more importantly because CNN requires a specific structured organization of the inputs, as in the case of images, which is not straightforward for omics data. In an attempt to overcome these limitations, we proposed an omics classification pipeline based on 1D CNN, which used dimensionality reduction methods to alleviate the high dimensionality issue of omics data and clustering techniques to organize the input features in a meaningful manner allowing the CNN method to utilize this feature organization to improve classification performance. 1D was preferred over the 2D CNN method because of the inherent organization of the omics data as 1D feature vectors.

The present study explored designing and applying a new CNN-based tool for the discovery of diagnostic biosignatures and models for Ischemic Stroke and COVID-19 using high-dimensionality transcriptomics and proteomics datasets, respectively. Regarding Ischemic Stroke, an early and accurate diagnosis can improve the probability of a positive outcome. The objective of many studies was to identify biomarkers to facilitate the early diagnosis of acute ischemic stroke (AIS). In a prior study [17], the most important genes were identified using differential expression analysis. These were tested in a logistic regression model and further validated by QRT-PCR. Another study that provided insight into the molecular of AIS [18], made use of a machine-learning technique known as genetic algorithm k-nearest neighbors’ (GA/kNN) to identify a pattern of gene expression that could optimally discriminate between patients and neurologically asymptomatic controls. Furthermore, a more recent study [5], handled a hybrid genetic algorithm–support vector machine learning tool combined with a network comparison approach to identify transcription patterns characteristic of patients with acute ischemic stroke. The approach in a recent study [19], was to identify the optimal model, by comparing different computational methods, for analyzing microRNA expression data for discriminating patients with AIS from controls. Machine learning algorithms, including artificial neural networks (ANNs), random forests, extreme gradient boosting, and support vector machines (SVM) were applied. The different models used three different combinations of microRNAs. ANNs and SVM models had the best performance, according to AUC (Area Under Curve). Moreover, concerning COVID-19, several recent studies have focused their interest on identifying emerging biomarkers for SARS-CoV-2 detection, COVID-19 diagnostics, treatment, prognosis, and the design of new therapies. Specifically, in a recent study, a combination of clinical parameters with protein abundancies was examined to identify the survival or death in COVID-19 patients [20]. For this analysis, the WEKA machine tool was used for training and validation with 9 clinical and 45 protein-based putative biomarkers being associated with the survival/death of COVID-19 patients. In another study examining the severity of the disease based on proteomic data [6], it was found that the Support Vector Machine had the best performance compared to other machine learning algorithms. Furthermore, a combination of Proteomic and Metabolomic data was used in another study to predict severity using the Random Forest model and reported an AUC of 95.7 % [21].

## 2 Methods

1) Datasets

Data of the first dataset were from microarray experiments (Affymetrix whole-genome expression arrays U133 2.0) on peripheral blood samples from stroke patients and from control non-stroke patients. The samples were derived from three different scientific studies. As the three datasets were generated using different instrumentation and experimental setup, they were separately normalized using the raw data to homogenize them generating a single expression matrix with a consistent scaling of expression levels. The dataset [9] consists of 20 stroke and control peripheral blood mononuclear cell (PBMC) samples, while [10] has 39 stroke and 25 control whole blood samples, that were evaluated at three different time points (within 3 h (h), 5, and 24 h of the stroke event); only the within 3 h time point data were used for this study. The [11] has whole blood for 23 stroke and control samples. The final integrated dataset included patients who had IS and healthy patients (control group). There was a total of 82 patients with IS and 68 healthy patients participating in the study. After keeping the expression values of commonly measured genes in all independent studies we ended up with 13243 quantified gene transcripts for each sample. A linear regression method was used to correct data for different sample types (PBMC and whole blood), while also transcripts significantly differentiated between the two sample types were filtered out. This study aimed to predict whether the sample would manifest a stroke, depending on the expression of its specific genes.

The samples of the second dataset were derived from the MGH study [12]. The data include protein measurements using a high throughput antibody technique and essential clinical parameters from plasma samples of COVID-19 patients and controls. The study was conducted by a group of clinicians and immunologists at MGH, which included patients with a clinical concern for COVID-19 upon Emergency Department arrival. Out of the 384 patients enrolled, 306 tested positive for COVID-19, whereas the 78, who tested negative for COVID-19, were included as a control group. The blood samples from the patients’ group were selected on days 0, 3, and 7, while virus-negative patients had sampling only on day 0. In the context of the present study, we used data obtained on day 0 to discriminate between COVID-19 and control patients. Several clinical and demographic characteristics were also collected alongside the proteomics measurements including age, body mass index (BMI), pre-existing medical conditions, and laboratory measurements of C-reactive protein (CRP), absolute neutrophil count, and D-dimer.

For each omics measurement of both datasets, logistic regression models were fitted using the sklearn version 1.1.1 Python package.

2) Omics-CNN

### 2.1 Data preprocessing

**Fig. 1:**
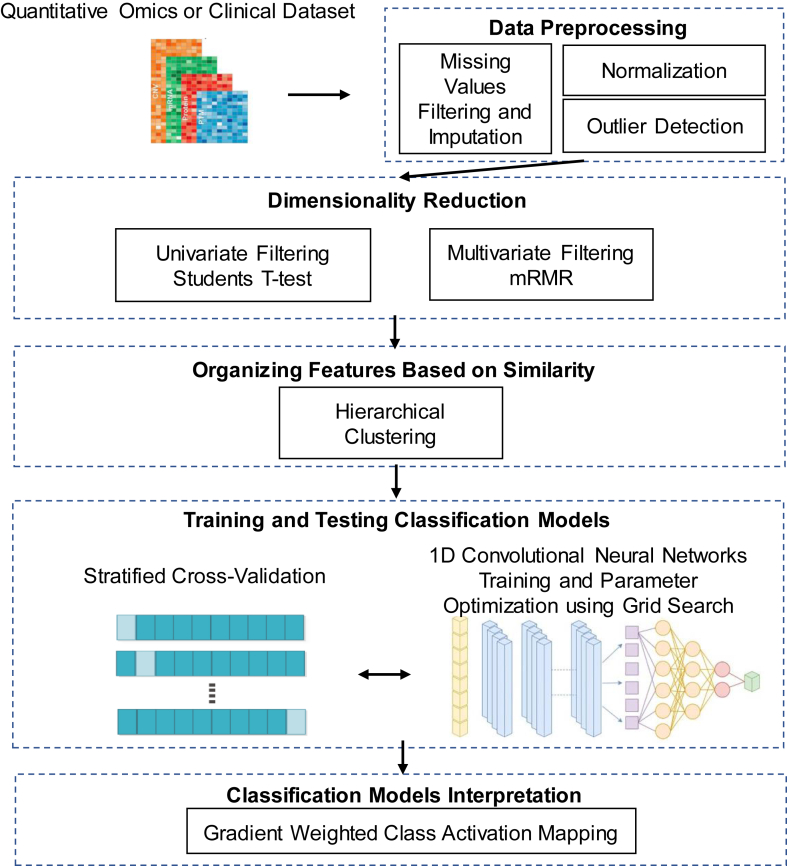
The Omics-CNN pipeline. Different colors in the input data correspond to omics data from different modalities showing that the Omic-CNN pipeline is applicable to all quantitative omics data. (For interpretation of the references to color in this figure legend, the reader is referred to the Web version of this article.)

The central idea of the study was to examine whether a previously established imaging data analysis deep learning method, namely the Convolutional Neural Networks (CNN), is suitable for high-throughput omics data analysis. To make CNN applicable to high-throughput omics data, we designed and implemented a new pipeline and tool called Omics-CNN. The proposed pipeline is presented in detail in Fig 1. The first step of the proposed pipeline was data preprocessing. The existence of missing values in the datasets was examined since data imputation or filtering techniques are essential for training CNN models. The attributes that had more than 30 % missing values were filtered. A kNN algorithm was used to impute the values for the remaining missing values, using the KNNImputer method of the sklearn library version 1.1.1, and k = 20 (default value) [13]. We used a combination of the Local Outlier Factor method [14] and Principal Components Analysis (PCA) to identify potential outliers. From both datasets, less than 5 % of the data were marked as outliers.

### 2.2 Dimensionality reduction methods

As a next step, dimensionality reduction techniques were applied to remove features that were not significant for each classification problem. The number of features, for each problem, was determined by a univariate technique, student's T-test, and a multivariate technique, the MRMR (Minimum Redundancy-Maximum Relevance) method [15] were both explored using the MIFS python package version: 0.0.1.0 [16]. Developing classification models using a large set of features may result in high computational resource requirements, limited accuracy and lack of reproducibility in validation datasets. However, for the identification of meaningful biomarkers, it becomes essential to restrict the number of features considered. The T-test method is commonly employed to identify features with a high correlation to the target variable. The Student's T-test is a parametric univariate filtering test requiring features to be normally distributed. Normality was verified on the logarithmized preprocessed features of both datasets using the Shapiro-Wilk method. Additionally, in a subsequent approach, the interaction between features in determining the outcome is taken into account using methods such as MRMR to uncover the combined influence of features on the final result. The MRMR feature selection process was conducted iteratively, varying the number of selected features for each iteration and testing a selection from 2 to 100 features using stratified 5-fold cross-validation. The number of features that yielded the best performance was determined as the optimal choice. Additionally, an experiment was conducted to determine the number of samples to use for kernel density estimation with the k-nearest neighbors (kNN) method. The library utilized for this purpose provided an estimation range between 3 and 10 samples with the selection of 7 neighbors returning the best performance and used throughout all our results.

### 2.3 Hierarchical clustering

The need for hierarchical clustering was identified due to the increase in the performance of classification models when using Hierarchical clustering compared to the performance obtained when not using any clustering. In the IS diagnosis dataset, the model without any clustering demonstrated s mean specificity of 90.99 % ( ± 8.83 %), mean accuracy of 94.00 % ( ± 4.4 %), and mean sensitivity of 96.4 % ( ± 2.94 %), but when hierarchical clustering was used, the mean specificity, mean accuracy, and mean sensitivity reached 96.92 % (±6.15 %), 98 % (±4%) and 98.82 (±2.35 %), respectively (Table 1).

**Table 1:** Comparing Results of different methods in Ischemic Stroke and Covid-19 datasets. The first approach utilizes Hierarchical Clustering combined with a 1D CNN (Convolutional Neural Network), where all features are used as input for the CNN. In the second approach, a T-test is performed on the features before Hierarchical Clustering, and the selected features are then fed into the 1D CNN. The third approach incorporates mRMR (Minimum Redundancy Maximum Relevance) Feature Filtering, followed by Hierarchical Clustering and subsequent analysis using the 1D CNN. For all the different dimensionality reduction setups, results without using hierarchical clustering are also provided. The reported performance results are averaged over 10 runs using 5-fold stratified cross-validation.

	Ischemic Stroke Diagnosis				Covid-19 Diagnosis			
Omics-CNN version	Accuracy	Sensitivity	Specificity	#Attributes	Accuracy	Sensitivity	Specificity	#Attributes
Omics–CNN–all Features	98.00 % (±4.00 %)	98.82 % (±2.35 %)	96.92 % (±6.15 %)	13243	96.72 % (±5.77 %)	82.50 % (±29.15 %)	98.87 % (±2.26 %)	1107
Omics–CNN–all Features-No Clustering	94.00 % (±4.40 %)	96.40 % (±2.94 %)	90.99 % (±8.83 %)	13243	93.76 % (±5.08 %)	82.25 % (±15.81 %)	96.11 % (±3.77 %)	1107
Omics–CNN–Univariate Filtering-Hierarchical Clustering	96.00 % (±4.90 %)	96.47 % (±4.71 %)	95.38 % (±6.15 %)	1911	95.41 % (±5.02 %)	75.28 % (±26.36 %)	98.49 % (±2.20 %)	640
Omics–CNN–Univariate Filtering-No Clustering	94.67 % (±6.53 %)	95.29 % (±6.86 %)	93.85 % (±8.97 %)	1911	94.43 % (±6.27 %)	72.50 % (±33.91 %)	97.75 % (±2.77 %)	640
Omics–CNN–mRMR Filtering-Hierarchical Clustering	74.53 % (±7.97 %)	75.59 % (±5.48 %)	72.86 % (±21.74 %)	15	86.89 % (±5.58 %)	27.78 % (±20.09 %)	96.17 % (±5.01 %)	14
Omics–CNN–mRMR Filtering-No Clustering	73.84 % (±9.71 %)	74.41 % (±8.15 %)	72.86 % (±21.74 %)	15	85.57 % (±4.80 %)	18.61 % (±11.44 %)	96.18 % (±5.02 %)	14

For the Covid-19 diagnosis, the model without any clustering demonstrated s mean specificity of 96.11 % ( ± 3.77 %), mean accuracy of 93.76 % ( ± 5.08 %), and mean sensitivity of 82.25 % ( ± 15.81 %), but when hierarchical clustering was used, the mean specificity, mean accuracy, and mean sensitivity reached 98.87 % (±2.26 %), 96.72 % (±5.77 %) and 82.50 (±29.15 %), respectively (Table 1).

Thus, the hierarchical clustering method was able to increase the classification metrics of the obtained classification models. The convergence times of both models were also reduced. Overall, the application of hierarchical clustering proved beneficial in optimizing the performance of the model for both datasets, facilitating the development of a unified and powerful computational tool suitable for biomarker detection.

So, sequences based on the similarity of the different attributes must be determined to correlate data before entering the Convolutional Neural Network [17]. The idea was to apply hierarchical clustering using the scipy. cluster.hierarchy package of the scipy version 1.8.1 [18]. The similarity between the attributes was determined by using the Euclidean distance metric. The clusters were merged by using the ward method which uses the square of the distances between the clusters. The dendrogram plot, generated using the dendrogram function from scipy.cluster.hierarchy, was carefully examined.

### 2.4 Training and testing classification models

The sequence of the attributes that are specified from hierarchical clustering is set as input in a 1D CNN [19]. To implement the Network, Tensorflow [20] (tensorflow_version: 2.8.0) was used. The tool was chosen, taking into account the overarching research objective of identifying plausible biomarkers that can be utilized for the detection and characterization of pathogens. The high complexity of the data relating to genes and proteins is essentially the use of a tool that can detect underlying relationships in the data. Omics data often encompasses essential local patterns or motifs that play a significant role in determining their functionality. Specifically, 1D CNN models address the challenge of extracting and analyzing these important patterns, by applying convolutional filters across the input data to capture and identify critical local features. Moreover, the problem which arises from the high dimensionality of the input data is effectively mitigated by using 1D CNN. The suitability of 1D CNN models for omic data analysis is further underscored by their successful applications in various domains. They have been extensively utilized in tasks such as DNA sequence classification, where the complexity of the data requires advanced techniques to derive valuable insights. The primary objective of the project was to develop a tool capable of effectively analyzing omics data. To accomplish this goal, the project adopted a consistent approach, employing similar patterns in the models used for analyzing both datasets. The aim was to ensure optimal performance for both datasets through a unified methodology. In the following subsections, we present in more detail the 1D CNN Network Topology that was inferred deploying grid-search optimization for each of the setups of the Omics-CNN tool: i) Omics–CNN–All features, ii) Omics–CNN–Univariate Filtering and iii) Omics–CNN–mRMR Filtering.

### 2.5 Omics–CNN–all features 1D CNN Network Topology

**Multiple Convolutional Layers.** The model incorporates three Conv1D layers one after another (64 filters with a kernel size of 10, 7, and 7, respectively) to extract hierarchical and increasingly complex features from the input omics data. By stacking multiple convolutional layers, the model can learn both low-level and high-level features, which enables it to capture more diverse and abstract information from the data. As for both models the first attempts had to do with an analysis, using all features, the experiments, with more convolutional layers led to the high complexity of the model (especially for the Ischemic Stroke dataset), with extended coverage time. The ideal number for kernel size was determined after experimentation and taking into account the complexity of the input data.

**Dropout Regularization**. Dropout layers are inserted after the first convolutional layer, before the first dense layer, and before the final dense layer. Dropout randomly sets a fraction of the input units to 0 during training, which helps prevent overfitting by reducing the reliance on specific features and encouraging the learning of more generalized representations. In this case, dropout regularization is applied to the convolutional and dense layers to improve the model's ability to generalize and make robust predictions on unseen data.

**Pooling Layers.** AveragePooling1D layers with a pool size of 2 are used after the first convolutional layer and after the second convolutional layer. Pooling layers downsample the input by summarizing nearby values, capturing the most salient features while reducing the dimensionality of the data.

**Dense Layers.** In the last stage, the model uses a fully connected layer that enables the model to learn complex relationships between the extracted features and determine the classification of the sample in each category. The model includes two dense layers with 500 and 250 units, respectively, after the last pooling layer. They integrate the learned representations from the previous layers and perform high-level abstractions, allowing the model to make predictions based on these abstractions. Finally, a dense layer with 134 units is added after the previous dense layers. The logic used to determine the number of nodes followed an informal method of halving them at each subsequent level.

**Output Layer.** The model ends with a dense layer with 2 units and a softmax activation function, to predict one of the possible classes. The softmax activation ensures that the output values represent the model's predicted probabilities for each class.

The model is compiled using the ‘sparse_categorical_crossentropy’ loss function, and ‘stochastic gradient descent’ optimizer (sgd). To prevent overfitting and save computational resources, an early stopping callback was set to 100 epochs. These parameters were also used for the training needs of the models that make use of feature extraction methods.

### 2.6 Omics–CNN–univariate filtering 1D CNN Network Topology

**Convolutional Layers.** Two 1D convolutional layers are added after the initial layers. Both layers have 64 filters, a kernel size of 10, a stride of 1, a ‘relu’ activation function, and ‘same’ padding. These additional convolutional layers help capture more complex patterns and features in the input data.

**Max Pooling Layer.** A max pooling layer is added after each convolutional layer with a pool size of 2. Max pooling reduces the spatial dimensions of the feature maps while preserving the most important features.

**Dense Layers.** The architecture includes two fully connected dense layers. The first dense layer has 500 units, and the second dense layer has 250 units. Both layers use the ‘relu’ activation function.

**Flatten Layer.** The flatten layer is used to convert the multidimensional feature maps into a 1D feature vector before connecting them to the dense layers.

**Output Layers**. The model ends with two dense layers. Before entering the final dense layer, a dense layer with 134 units with the ‘relu’ activation function. The final dense layer has 2 units with the ‘softmax’ activation function.

### 2.7 Omics–CNN–MRMR filtering 1D CNN Network Topology

**Convolutional Layer.** The first layer is a 1D convolutional layer with 64 filters, a kernel size of 10, and a ReLU activation function. The use of one extra Convolutional Layer was impossible as it led to extremely small vectors and the model couldn't classify the data.

**Dropout Layer.** A dropout layer is applied after the convolutional layer with a dropout rate of 0.2.

**Max Pooling Layer.** A max pooling layer with pool size 2 is applied to reduce the spatial dimensions of the feature maps while retaining the most important features.

**Dense Layers.** Two fully connected dense layers follow the pooling layer with 500 and 250 units, respectively.

**Flatten Layer.** A flatten layer is used to convert the multidimensional feature maps into a 1D feature vector.

**Dropout Layer.** Another dropout layer is added after flattening with a dropout rate of 0.2, further enhancing regularization.

**Output Layers.** Two dense layers with 134 and 2 units, respectively, are added to produce the final classification output. The last layer uses the softmax activation function.

The training and evaluation of the model are done by applying stratified k-fold cross-validation with k = 5, by using StratifiedKFold, from sklearn. This approach is selected to ensure the random separation of samples into folds. Also, the limited number of available samples in the datasets necessitates the use of this technique to avoid the overfitting of the model. The collection of data using DNA microarrays is known to be prohibitively costly, making it infeasible to employ them for large-scale patient studies. The performance of the model is evaluated by running 10 executions for each model and calculating averages and standard deviations of the reported metrics.

The Supplementary Table 1 presents the set of libraries and the versions used for the implementation of Omics-CNN.

### 2.8 Classification models interpretation

The use of CNN in high-dimensional omics data also aims to extract explainable classification rules from the trained models in line with the Explainable Artificial Intelligence (XAI) concept. For that reason, the Gradient Weighted Class Activation Mapping (Grad-CAM) method was applied with this method being able to highlight the most important areas of the input vector, which lead to the classification into each of the two categories.

The proposed pipeline was developed in the Python programming language (python version 3.9), was deposited to GitHub, and is publicly available at *Omics-CNN (*https://github.com/alphazita/Omics-CNN*).*

Α Graphical representation of the proposed pipeline is presented in Fig. 1. In the first stage, the data are preprocessed, to filter samples with more than 30 % missing values, and KNN impute algorithm is performed to impute the remaining ones. Then, dimensionality reduction was performed using MRMR and two-sided independent samples Student's T-test). Hierarchical clustering is applied to the remaining data to group and order the attributes according to their similarity, before the processing of the Convolutional Neural Network. In the final stage, after network training, Gradient Weighted Class Activation Mapping is performed to identify the most valuable attributes for the classification of the sample in each of the classes.

## 3 Results

**Fig. 2:**
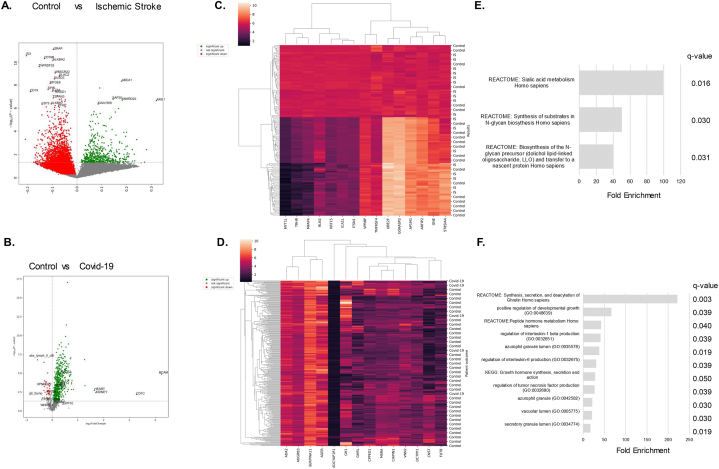
A. Differential expression analysis (volcano plot) between Ischemic Stroke (IS) and control using Student's t-test. Significantly upregulated molecules are presented in green and significantly downregulated molecules in red with a p-value threshold of 0.05 being used to infer significance. B. Differential expression analysis (volcano plot) between COVID-19 patients and controls using Student's t-test. Significantly upregulated molecules are presented in green and significantly downregulated molecules in red with a p-value threshold of 0.05 being used to infer significance. C. Heatmap and hierarchical clustering of selected biosignature for IS diagnosis. D. Heatmap and hierarchical clustering of selected biosignatures for COVID-19 diagnosis. E. Pathway enrichment analysis of IS differentially expressed genes. F. Pathway enrichment analysis of COVID-19 differentiated proteins. (For interpretation of the references to color in this figure legend, the reader is referred to the Web version of this article.)

From the volcano plots, in Fig. 2A and **B**, we confirm the presence of discriminative features for both datasets. Taking as a reference the patients’ groups, we can see from the plots, that when we compare patients with Ischemic Stroke with the Control Group, there are more downregulated molecules. When it comes to the COVID-19 problem, more attributes are significantly upregulated in patients with COVID-19. The difference in the expression levels as it emerges by the application of the Student T-test provides useful knowledge regarding the expression of the attributes in the different groups. The heatmaps visualize the z-score transformed expression levels of the selected Minimum Redundancy Maximum Relevance (mRMR) features across all the samples. These diagrams provide an overview of the samples in identifying whether value fluctuations in their attributes play a crucial role in the classification of the sample. The highlighted as most valuable from the MRMR method, are having less redundancy by design and are expected to be independently essential for the classification of the samples with minimum mutual information among them. From the two heatmaps (Fig. 2C and **D**), it is noted that some of the selected features still present common expression patterns despite the MRMR dimensionality reduction. Fig. 2E and **F** presents the pathway and functional enrichment analysis of the revealed biosignatures for ischemic stroke and COVID-19 against KEGG, Reactome, and Gene Ontology terms using the David web tool [21]. Functional and pathway enrichment analysis pinpointed the importance of the sialic acid metabolism pathway for ischemic stroke diagnosis and several functional terms for the Covid-19 diagnosis with the most important one being the pathway of synthesis, secretion, and diacylation of Ghrelin Homo Sapiens.

The datasets obtained from the above-mentioned preprocessing pipeline were used to assess the efficiency of the model. The present manuscript explores different model architectures by differentiating the hyperparameters.

**Fig. 3:**
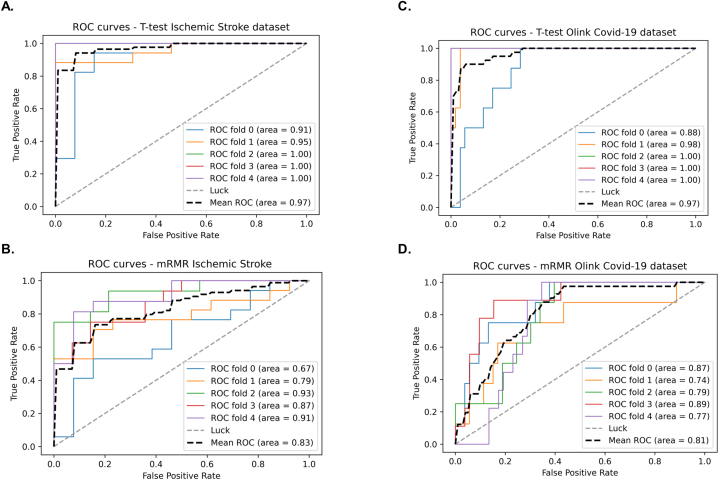
A. ROC curves of Omics-CNN on Ischemic Stroke diagnosis using Student's T-test for dimensionality reduction. B. ROC curves of Omics-CNN on Ischemic Stroke diagnosis using mRMR for dimensionality reduction. C. ROC curves of Omics-CNN on Covid-19 diagnosis using Student's T-test for dimensionality reduction. D. ROC curves of Omics-CNN on Covid-19 diagnosis using mRMR for dimensionality reduction.

Each method has its limitations and strengths specific to the type of application, as the data are reduced through the different methods including a representative univariate filtering method (Student's t-test) and a multi-variate one (MRMR). Table 1 shows accuracy, sensitivity, specificity, and accuracy for the two datasets and the different methods as an average of 10 runs of the overall analysis. Logistic regression methods using each feature separately were not able to return accuracy bigger than 65 % for any feature for both datasets. The results of Table 1 show that Omics-CNN significantly outperformed univariate logistic regression models demonstrating high sensitivity, specificity, and mean accuracy of the different folds of 5-Fold Stratified Cross-Validation. The best performance metrics were reported when all attributes were used without dimensionality reduction (98 % accuracy for Ischemic Stroke Diagnosis and 96.72 % accuracy for COVID-19 diagnosis), but the loss of accuracy when using univariate filtering was marginal making it the most suitable method. When the multivariate method MRMR was used a significant accuracy drop was observed but the number of revealed attributes also substantially dropped. It is noteworthy, that the use of hierarchical clustering improved the performance of all different versions of Omics-CNN (∼1–6%). The uplift in accuracy as expected was bigger when applied to the Omics-CNN version without feature selection because of the strongest associations between the selected features. In contrast, the uplift from using hierarchical clustering was marginal for both datasets when using multivariate dimensionality reduction because of the lack of redundancy and mutual information among the selected features which reduces the impact of clustering in the overall method. The behaviour of the different folds during the training of the model is shown in the ROC curves of Fig. 3 for the models developed using hierarchical clustering and of Supplementary Fig. 1 for the models developed without using hierarchical clustering.

When the Gradient Weighted Class Activation Mapping analysis was performed on the CNN models retrieved when using univariate filtering (students Τ-test) for dimensionality reduction, it was shown that the significant genes are more dispersed in the IS diagnosis dataset while the significant proteins for the Covid-19 diagnosis were accumulated on one of the clusters revealed from the hierarchical clustering (red cluster) confirming the multifactorial nature of the IS in contrast to Covid-19 which is attributed to a pathogenic coronavirus. It is noteworthy, that for the Covid-19 diagnosis only four (ADGRB3, VNN2, AGER, CIAPIN1) out of the fourteen proteins selected from mRMR were in the significant cluster of proteins revealed from feature significance analysis on the univariate filtering-based selected proteins. This was expected since mRMR tries to decrease the number of redundant features from the same cluster but also confirms the significance of VNN2 in the diagnosis of COVID-19.

**Fig. 4:**
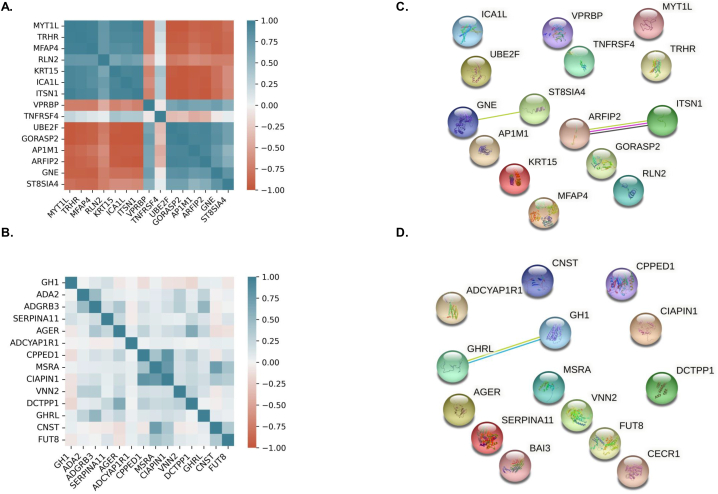
A. Spearman's correlation and hierarchical clustering of the revealed IS biosignature. B. Spearman's correlation and hierarchical clustering of the revealed COVID-19 biosignature. C. Reconstructed protein-protein interaction network using StringDB [22] for the revealed IS biosignature. D. Reconstructed protein-protein interaction network using StringDB [22] for the revealed COVID-19 biosignature.

Fig. 4A and **C** presents Spearman's correlation and hierarchical clustering of the revealed ischemic stroke biosignature and their corresponding reconstructed protein-protein interaction network using StringDB [22]. The same results are provided also for the Covid-19 diagnostic biosignature in Fig. 4B and D. This analysis showed that the MRMR dimensionality reduction for stroke returned a biosignature composed of 2 highly correlated clusters and two uncorrelated proteins while only weak correlations are presented among the proteins of the COVID-19 diagnostic biosignature. PPI networks showed only very few interactions among the revealed potential biomarkers suggesting that they correspond to different molecular mechanisms and justifying their presence in the revealed biosignatures.

Supplementary Tables 2 and 3 present the contribution of each of the revealed features in the classification of each one of the diagnostic problems in the form of the MRMR score. Gradient Weighted Class Activation Mapping analysis was finally performed to explore the contribution of each one of the markers in the classification decision for each one of the prediction problems. For the Ischemic Stroke dataset, as presented in the Supplementary Fig. 2 graph, the most significant contributing transcripts were: KRT15 (Keratin 15), VPRBP (Viral protein R binding protein), TNFRSF4 (TNF receptor superfamily member 4), GORASP2 (Golgi reassembly stacking protein 2). while the most significant contributing protein for COVID-19 diagnosis was VNN2 (vanin 2).

## 4 Discussion

The present manuscript introduces a CNN-based model applicable for biomarkers discovery and classification model development using omics data. We aimed to design a methodology based on Convolutional Neural Networks for the analysis of high-throughput omics data. CNNs were selected due to their ability to identify correlations between input elements and utilize them to build generalizable classification models. This tool is traditionally used for image classification. This property of CNNs allows modeling the complex and non-linear associations of omics data with disease diagnosis. The 1D CNNs were selected because the application domain of the developed tool is omics data analysis with omics data being organized in one-dimensional feature vectors. Using 1D CNN enabled capturing temporal relationships between values in the input, while coupling it with dimensionality reduction and clustering techniques made CNN applicable for the classification of the omics dataset. Moreover, the use of the dimensionality reduction part of the method as well as a combination of pre and post-processing methods for explaining the trained CNN models allowed for making useful scientific conclusions.

Omics-CNN was applied in two classification problems using high-throughput omics data. The first dataset was a transcriptomic dataset that was used [6] for training and testing diagnostic models for ischemic stroke. In a previous application, this dataset had been analyzed with a hybrid genetic algorithm–support vector machine learning tool for the identification of transcription patterns characteristic of patients with ischemic stroke and the diagnosis of ischemic stroke [6]. Moreover, the Massachusetts General Hospital (MGH) COVID-19 study dataset was used to develop diagnostic models and biosignatures for COVID-19 using proteomics and clinical features of the patients. The initial analysis of the MGH COVID-19 study investigated associations between plasma ACE2 and the outcome of COVID-19 [12]. This study used non-parametric statistical tests for group comparison. In a subsequent study, more than 200 proteins were found to be significantly elevated at infection and many of these are related to cytokine response and other immune-related functions [13].

For both applications, diagnostic models with higher AUC (Area Under Curve) compared to existing models were developed. Three different versions of Omics-CNN were used without using dimensionality reduction, using univariate feature filtering and using multivariate feature filtering method. The hierarchical clustering component was found to increase the performance of all three versions of Omics-CNN but as expected the improvement was higher when using all features because of the higher presence of mutual information among the genes which makes the clustering approach more relevant. For both applications, diagnostic models with higher AUC (Area Under Curve) compared to existing models were developed when using all features showing the ability of 1D CNN to handle high dimensional data without suffering from the curse of dimensionality. In opposite, using the dimensionality reduction techniques enabled the identification of highly interpretable biosignatures making the overall pipeline more meaningful for translational applications. In the stroke diagnosis problem, after experimentation in model architecture, a biosignature of 15 genes with an AUC cross-validation performance of 83 % was identified, as reported in Fig. 3B. Respectively, in the COVID-19 problem, a biosignature of 14 genes was discovered, with an AUC cross-validation performance of 81 % (Fig. 3D). Higher classification metrics were returned when using univariate filtering compared to using multivariate filtering opposite to what is expected when other more conventional classification models are used [23]. This might be explained by the ability of CNNs to deal with input data of high redundancy and from the fact that in the proposed pipeline these features are provided as inputs in a meaningful order into the classification model enabling it to identify and utilize hidden patterns in the data. The classification performance of the proposed method in Ischemic Stroke diagnosis, when using univariate filtering substantially, exceeded the previously reported classification performances on the same dataset of a Random Forest-based approach [24] and embedded dimensionality reduction and classification method [6], increasing cross-validation performance from 82.07 % to 89.57 % respectively to 96 %. Similar results are not available for the COVID-19 dataset since it has not been previously directly used for COVID-19 diagnosis but only for COVID-19 severity. However, it is important that the proposed CNN-based tool was able to substantially improve the diagnostic accuracy compared to single biomarkers demonstrating its suitability for a precision medicine approach for the diagnosis of multifactorial diseases. The application of machine learning for discovering diagnostic biosignatures for COVID-19 is significant for improving the diagnostic performance of existing methods (e.g. qPCR) at an early stage and promoting our understanding of the pathophysiological mechanisms involved in the disease [25].

Even though the deployed method required a high number of features to maximize the accuracy in diagnosing Ischemic Stroke and COVID-19, a combination of multi-variate filtering (mRMR) and explainable machine learning techniques (Gradient Weighted Class Activation Mapping) allowed the discovery of interpretable biosignatures and shed light into the most significant features and their contribution to the disease diagnosis. Regarding IS, the revealed biosignature was associated with the sialic acid metabolism pathway, a novel mechanism activated after stroke [26] and a potential therapeutic target for atherosclerosis-related diseases in general [27]. The most significantly contributing transcripts for stroke diagnosis were KRT15 (Keratin 15), VPRBP (Viral protein R binding protein), TNFRSF4 (TNF receptor superfamily member 4), GORASP2 (Golgi reassembly stacking protein 2), with only TNFRSF4 being previously associated with stroke in genetic level [28,29] and GORASP2 being indirectly suggested as potential therapeutic targets for ischemic injury in the Golgi apparatus [30]. Regarding the Covid-19 diagnosis, the revealed biosignature was associated with the synthesis, secretion, and diacylation of Ghrelin, with the Ghrelin enzyme being previously associated with SARS-CoV2 infection [31] and its activity in the stomach and weight regulation [32]. Moreover, the revealed Covid-19 diagnostic biosignature was enriched with terms associated with the regulation of interleukin-1 and interleukin-6 which involve the most widely studied mechanism for Covid-19 diagnosis [33]. However, the most significantly contributing protein in the diagnostic model was not one of the known markers but VNN2 (Vanin 2). According to the Human Protein Atlas [34], Vanin-2 is a protein mostly expressed in bone marrow, lymphoid tissues, and respiratory tissues and is involved in the metabolism and metabolism of water-soluble vitamins and cofactors. Vanin-2 has not been extensively studied as a diagnostic marker for Covid-19 but a previous study has shown that treating Covid-19 patients with Mesenchymal stem cells leads to mobilizing a subpopulation of hematopoietic stem/progenitor-like cells with increased expression of VNN2 showing a potential mechanism that might be linked with the upregulation of VNN2 in Covid-19 patients and its diagnostic potential.

Despite the promising results of the proposed method in the present manuscript, our study possesses certain limitations. First, only a limited set of some of the most widespread filtering dimensionality reductions was deployed. Other more advanced feature extraction methods, such as NormLime and genetic algorithms, might perform even better when integrated into the Omics-CNN pipeline. Moreover, the OmicsCNN tool demonstrated great ability in combining high dimensionality single omics datasets but results could improve even better when using multi-omics datasets. Furthermore, the revealed biosignatures and predictive models for both applications should be validated in independent testing datasets to explore their potential value for application in translational research applications. Even though the exploitation of such big biosignatures in translational applications is very difficult, modern advances in transcriptomics and proteomics, such as the availability of the highly reproducible Olink® Explore 1536 platform, which can quantify 1536 proteins and aptamer-based commercial panels [35], are a significant step towards the translational applicability of diagnostic models based even on thousands of biomarkers.

In summary, the present work paved the way for the utilization of a useful tool in a very wide range of data. It introduced the application of Convolutional Neural Networks for omics data classification. Depending on the needs of each user, each of the revealed classification models from each run of the Omics-CNN tool, using all features selected with multivariate filtering, can be used as a tool for disease diagnosis. At the same time, subsets of features with or without overexpression in the samples, or correlations between the features can be identified. An extension of the methodology of the present work would be the combination of different types of data in different stages of processing of the Convolutional Neural Network. The idea of the present work was developed based on the philosophy that the machine learning model is not seen as a black box but rather as a tool in which the user can determine how the model reaches a specific decision.

## Author contribution statement

Anastasia Zompola: performed the experiments; analyzed and interpreted the data; wrote the paper.

Konstantinos Theofilatos, Aigli Korfiati and Seferina Mavroudi: conceived and designed the experiments; contributed reagents, materials, analysis tools or data; analyzed and interpreted the data.

## Data availability statement

Publicly available data is used.

## Funding and competing interests

Dr Konstantinos Theofilatos has been supported by the PG/20/10387 10.13039/501100000274British Heart Foundation project grant. The authors declare no conflict of interest.

## Declaration of competing interest

The authors declare that they have no known competing financial interests or personal relationships that could have appeared to influence the work reported in this paper.
