# Supplementary material for: Omics-CNN: A comprehensive pipeline for predictive analytics in quantitative omics using one-dimensional convolutional neural networks
Source: Heliyon. 2023 Oct 28;9(11):e21165. doi: 10.1016/j.heliyon.2023.e21165 (PMC10658203; doi:10.1016/j.heliyon.2023.e21165)
Supplement: Multimedia component 1 [file mmc1.docx]

**Supplementary material for the article:**

***Omics-CNN: a comprehensive pipeline for predictive analytics in quantitative omics using one dimensional Convolutional Neural Networks***

Anastasia Zombola^1^, Aigli Korfiati^2^, Konstantinos Theofilatos^3^, Seferina Mavroudi^2,4^

*^1^ Department of Electrical and Computer Engineering, University of Patras, Patra, Greece,*

*^2^InSyBio PC, Patras Science Park, Patra, Greece*

*^3^King’s British Heart Foundation Centre, Kings College London, United Kingdom*

*^4^Department of Nursing, School of Rehabilitation Sciences, University of Patras, Patra, Greece*

**Supplementary Figures**


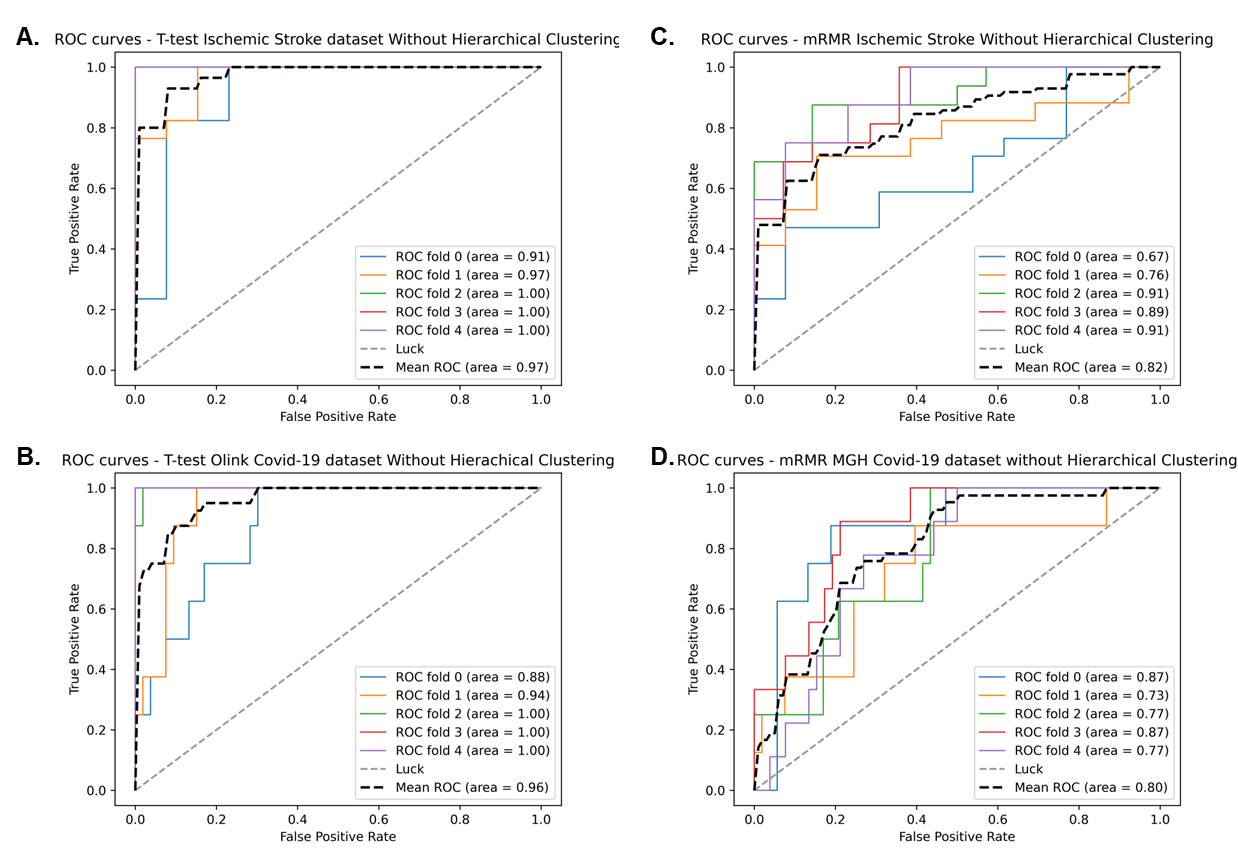


**Supplementary Figure 1.** A. ROC curves of Omics-CNN on Ischemic Stroke diagnosis using Student’s T-test for dimensionality reduction without hierarchical clustering. B. ROC curves of Omics-CNN on Covid-19 diagnosis using Student’s T-test for dimensionality reduction without hierarchical clustering. C. ROC curves of Omics-CNN on Ischemic Stroke diagnosis using mRMR for dimensionality reduction without hierarchical clustering. D. ROC curves of Omics-CNN on Covid-19 diagnosis using mRMR for dimensionality reduction without hierarchical clustering


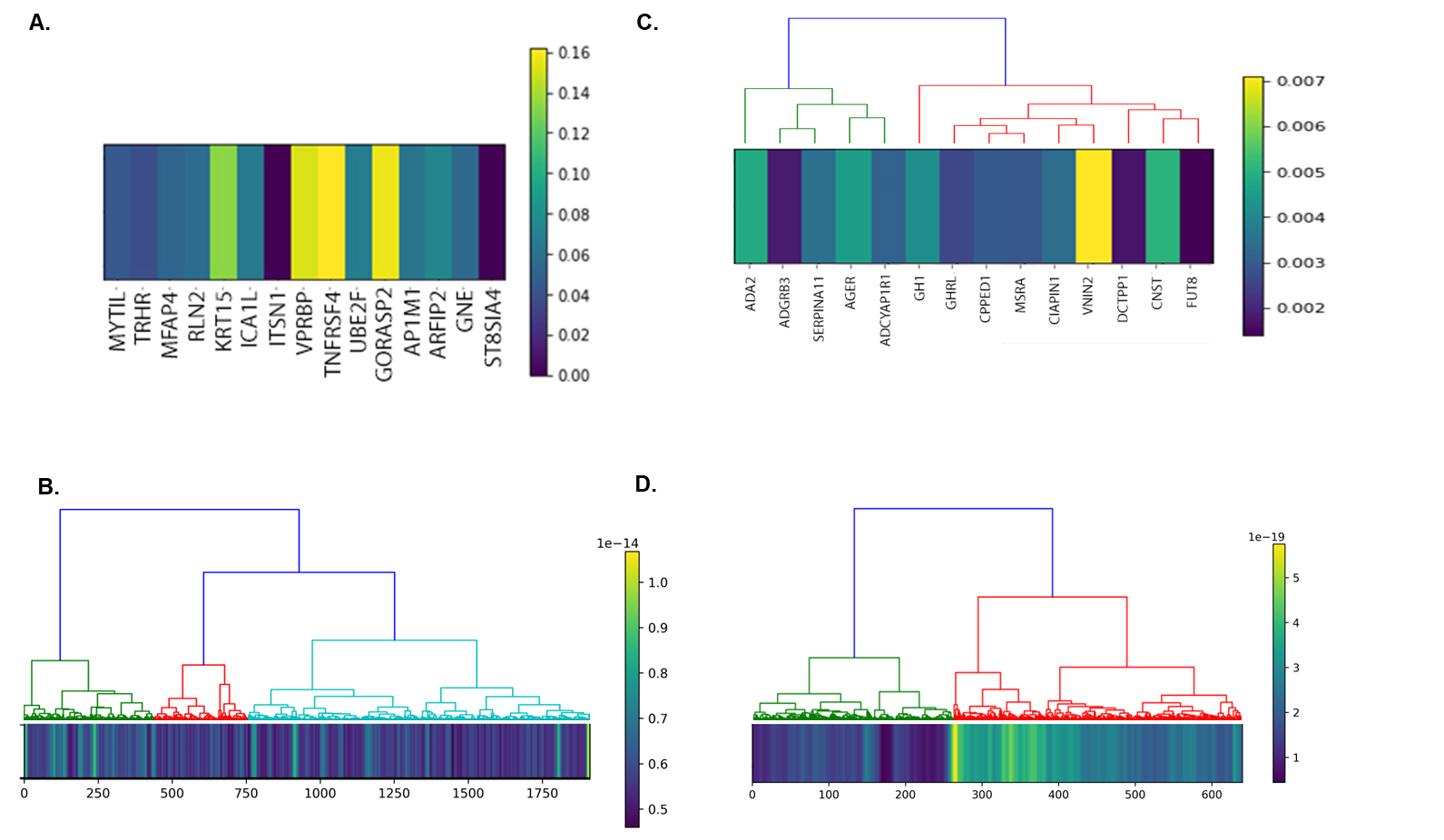


**Supplementary Figure 2.** A. Gradient Weighted Class Activation Mapping and Dendrogram of attributes selected from T-Test in Ischemic Stroke data. B. Gradient Weighted Class Activation Mapping and Dendrogram of attributes selected from mRMR in Ischemic Stroke data. C. Gradient Weighted Class Activation Mapping and Dendrogram of attributes selected from T-Test from MGH Covid-19 dataset. D. Gradient Weighted Class Activation Mapping and Dendrogram of attributes selected from mRMR from Olink Covid-19 diagnosis dataset

**Supplementary Tables.**

| **Library** | **Version** |
| --- | --- |
| **sklearn** | 1.1.1 |
| **MIFS python package** | 0.0.1.0 |
| **scipy** | 1.8.1 |
| **TensorFlow** | 2.8.0 |

**Supplementary Table 1:** Libraries and tools used for the implementation of Omics-CNN tool.

| Attributes | Gene Symbols | mRmr scores |
| --- | --- | --- |
| ubiquitin conjugating enzyme E2 F | 'UBE2F' | 0.2740 |
| Viral protein R binding protein | 'VPRBP' | 0.0543 |
| thyrotropin releasing hormone receptor | 'TRHR' | 0.013 |
| glucosamine (UDP-N-acetyl)-2-epimerase/N-acetylmannosamine kinase | 'GNE' | 0.0196 |
| Relaxin 2 | 'RLN2' | 0.0316 |
| Adaptor-related protein complex 1 subunit mu 1 | 'AP1M1' | 0.0250 |
| myelin transcription factor 1 like | 'MYT1L' | 0.0348 |
| islet cell autoantigen 1 like | 'ICA1L' | 0.0157 |
| ST8 alpha-N-acetyl-neuraminide alpha-2,8-sialyltransferase 4 | 'ST8SIA4' | 0.0023 |
| microfibril associated protein 4 | 'MFAP4' | 0.0217 |
| intersectin 1 | 'ITSN1' | -0.0080 |
| TNF receptor superfamily member 4 | 'TNFRSF4' | -0.0064 |
| Keratin 15 | 'KRT15' | -0.0053 |
| ADP ribosylation factor interacting protein 2 | 'ARFIP2' | -0.0012 |
| Golgi reassembly stacking protein 2 | 'GORASP2' | -0.0021 |

Supplementary Table 2: Selected attributes from Ischemic Stroke data with the mRMr method.

| Attributes | Gene Symbols | mRmr scores |
| --- | --- | --- |
| adenosine deaminase 2 | 'ADA2' | 0.1255 |
| serpin family A member 11 | 'SERPINA11' | 0.0220 |
| adhesion G protein-coupled receptor B3 | 'ADGRB3' | 0.0046 |
| methionine sulfoxide reductase A | 'MSRA' | 0.0131 |
| vanin 2 | 'VNN2' | 0.0175 |
| calcineurin like phosphoesterase domain containing 1 | 'CPPED1' | 0.0011 |
| consortin, connexin sorting protein | 'CNST' | 0.0016 |
| advanced glycosylation end-product specific receptor | 'AGER' | 0.0018 |
| dCTP pyrophosphatase 1 | 'DCTPP1' | -0.0023 |
| cytokine induced apoptosis inhibitor 1 | 'CIAPIN1' | -0.0009 |
| fucosyltransferase 8 | 'FUT8' | -0.0124 |
| ADCYAP receptor type I | 'ADCYAP1R1' | -0.0111 |
| ghrelin and obestatin prepropeptide | 'GHRL' | -0.0074 |
| growth hormone 1 | 'GH1' | -0.0065 |

Supplementary Table 3: Selected attributes from MGH Covid-19 dataset with mRMR method
